# Spatiotemporal patterns and propagation of meteorological and hydrological drought in a humid basin of Southeast China

**DOI:** 10.1038/s41598-025-17005-1

**Published:** 2025-08-28

**Authors:** Beibei Gan, Meibing Liu, Hongming Cui, Xingwei Chen, Ying Chen, Lu Gao, Haijun Deng

**Affiliations:** 1 Institute of Geography, Fujian Normal University, Fuzhou, 350117 China; 2 School of Geographical Sciences, School of Carbon Neutrality Future Technology, Fujian Normal University, No.8 Shangsan Rd., Cangshan District, Fuzhou, 350007 China; 3 State Key Laboratory for Subtropical Mountain Ecology of the Ministry of Science and Technology and Fujian Province, Fujian Normal University, Fuzhou, 350007 China; 4 Fujian Provincial Engineering Research Center for Monitoring and Assessing Terrestrial Disasters, Fujian Normal University, Fuzhou, 350007 China

**Keywords:** Meteorological drought, Hydrological drought, Drought propagation, Influential factors, Climate sciences, Hydrology

## Abstract

Droughts have caused great damage due to climate change and intensified human activities. Quantifying the propagation law of meteorological drought (MD) to hydrological drought (HD) is essential for drought warning, defense and adaption. In this study, based on the standardized precipitation evapotranspiration index (SPEI) and standardized runoff index (SRI), we analyzed the spatiotemporal characteristics and propagation patterns of MD and HD 1975–2020 in Minjiang River Basin (MRB) located in Southeast China. Furthermore, the effects of climate change and large reservoirs on the spread of drought were discussed. The results indicated that MRB experienced relatively severe MD and HD after 2000. All subbasins experienced MD and HD in spring, while they exhibit a trend of hydrological humidification in winter. The drought propagation time (DPT) from MD to HD generally 3 months. And spring and summer generally have a shorter DPT (2 months) than autumn and winter (3–5 months). Precipitation is the key meteorological factor affecting drought conditions in all four seasons, contributing over 80% to MD and HD, while the total impact of temperature and actual evapotranspiration (ETa) is less than 20%. ENSO is the most influential circulation factor affecting the drought variability of MRB, contributing over 50% to MD and HD, and followed by PDO. Reservoir discharge is the key factor leading to the trend of hydrological humidification in autumn and winter. The operations of large reservoirs since 1996 have extended the DPT by 4, 2, 3, and 5 months in spring, summer, autumn, and winter, respectively.

## Introduction

Global warming and intensive human activities have changed the hydrological cycle greatly, resulting in frequent extreme events such as heatwaves, floods and droughts^1^. As one of the most severe natural disasters, drought seriously affects the ecological hydrological system and human society^2^. Drought can be broadly categorized into meteorological drought (MD), agricultural drought (AD), hydrological drought (HD) and socioeconomic drought (SED)^3^. MD occurs first in natural conditions. Prolonged MD can reduce soil moisture and runoff, while insufficient or unreplenished water resources may lead to HD^4^. HD is considered the most critical type of drought, which has threatened water supply safety and caused enormous economic losses^4,5^. Although drought is more common in arid and semi–arid regions, in recent years, humid regions affected by monsoon climate have also suffered seasonal drought frequently^6^. Therefore, understanding the spatiotemporal patterns and propagation mechanisms of MD and HD in humid areas is crucial for water resource management and drought mitigation.

The propagation from MD to HD can manifest as lagging, attenuation, prolongation, and spatial shifts^7^. In recent years, numerous studies have established linear or nonlinear relationships between different types of droughts to analyze drought propagation, using methods such as Pearson correlation, wavelet analysis, Bayesian network model and hydrological model^7,8^. The Pearson correlation method can be used to assess the monotonic relationship between two variables, regardless of whether the relationship is linear or nonlinear^9^. Sadhwani and Eldho^8^ emphasized that the Pearson correlation analysis provided good results in evaluating drought propagation in humid regions. Additionally, there are also spatial differences in drought propagation time (DPT). In general, areas with a humid climate showed a strong propagation relationship between meteorological and hydrological drought with shorter DPT, while arid climate areas showed a poorer relationship with longer DPT^10,11^. The propagation from MD to HD also has significant seasonal characteristics. In spring and summer with abundant precipitation, the DPT is usually shorter than that in autumn and winter^12^. However, with the global warming trend, rising temperatures will lead to more actual evapotranspiration and less runoff recharge, which further prolong the duration of drought in most regions of the world, including humid areas^13^.

Drought evolution and propagation characteristics are influenced by various factors, including climate change, watershed characteristics, and human activities^14^. Climate change (related to precipitation, temperature, evapotranspiration, etc.) is a key factor affecting the DPT^15^. Large–scale circulation factors such as the El Niño–Southern Oscillation (ENSO) and the North Atlantic Oscillation (NAO) influence convective meteorological processes by regulating sea surface temperatures or atmospheric circulation anomalies, thereby affecting the hydrological cycle and causing drought^16^. However, recent studies on drought causality emphasize that the occurrence and evolution of severe drought have shifted from purely natural drivers to a combination of climate change and human activities, such as irrigation, water extraction, and large hydraulic projects^17,18^. Large reservoirs redistribute runoff through water storage, leading to exacerbation or alleviation of HD in downstream areas^18^. The impact of reservoir operation on HD varies across different regions and watersheds, potentially extending^19^ or shortening the DPT^20^depending on their objectives and operational strategies^21^.

China is one of the countries affected by droughts seriously, with extreme drought events becoming more and more frequent^22^. At present, the spatiotemporal pattern of drought in China is undergoing continuous changes. The climate in the arid regions of northern China has exhibited a warming and wetting trend, while the long-term humid southern regions have experienced frequent seasonal droughts^23^. Therefore, it is of great significance to deeply study the characteristics of drought and its influential factors in humid regions and to take appropriate measures to mitigate the impacts of drought. In the past 58 years, the frequency of MD and HD has been the highest in the humid coastal areas in southeastern China^24^. However, prior drought research has mainly concentrated on arid and semi-arid regions, while the issue of quantifying the characteristics of drought propagation from meteorological drought to hydrological drought has been scarcely addressed in existing studies. Further understanding is needed on the impact of climate change and human activities, such as reservoir regulation, on the spread of drought in humid areas. It is essential to study the drought propagation characteristics and the effects of climate and reservoirs on drought in humid basins. Therefore, we selected the Minjiang River Basin (MRB) in the humid coastal region of Southeast China as the study area. The MRB is an important water conservation watershed, and its hydrological process is significantly affected by reservoir operations^25^. Seasonal drought events occur frequently, placing a serious threat to the water security of 12 million people in the watershed. The main objectives of this study were to: (1) analyze the spatiotemporal evolution of MD and HD in the MRB based on standardized indices; (2) investigate the drought propagation characteristics in different seasons and subbasin scales by calculating the DPT; and (3) explore the effects of climate change and human activities (reservoir operations) on the DPT.

## Materials and methods

### Study area

**Fig. 1:**
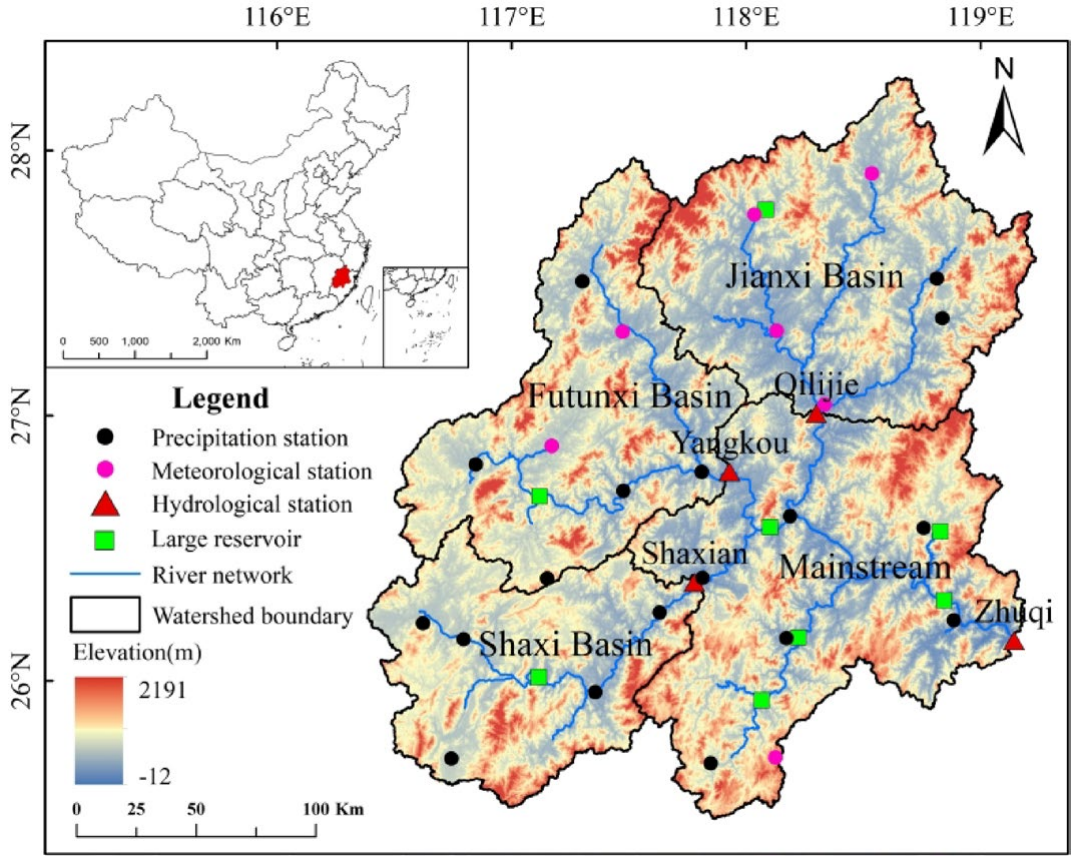
Location of the MRB and the distribution of observation stations and large reservoirs.

The MRB is located in Fujian Province, Southeast of China (116° 23’–119° 35’E, 25° 23’–28° 16’N), covering an area of 6.1 × 10^4^ km^2^. It accounts for nearly half of the area of Fujian Province (Fig. 1). The basin is stepped from the northwest to southeast, with three major tributaries (Jianxi, Futunxi, and Shaxi), eight large reservoirs. Three of the eight large reservoirs are located in Jianxi, Futunxi, and Shaxi, respectively, and the remaining five are in the mainstream. The annual average precipitation and temperature in the MRB are 1738 mm and 18 °C, respectively. Approximately 70% of the annual precipitation occurs in spring and summer, while 30% occurs in autumn and winter. The area is mainly affected by Meiyu from April to June, and typhoon storms from July to September. The absence of a typhoon in summer can reduce precipitation by 50% compared to the same historical period and result in continuously high temperatures. Due to the uneven spatiotemporal distribution of precipitation and the regulation of large reservoirs, seasonal drought events occur frequently and can seriously threaten the water supply in the downstream region.

### Datasets

#### Runoff and meteorological data

The monthly runoff data from 1975 to 2020 were selected from four hydrological stations located in Shaxian, Yangkou, Qilijie, and Zhuqi. The data were derived from the Fujian Hydrology and Water Resources Survey Center. Precipitation data of 18 precipitation stations and temperature data of 7 meteorological stations from 1975 to 2020 were derived from the National Meteorological Information Center of the China Meteorological Administration (CMA). Figure 1 shows the locations of the precipitation, meteorological, and hydrological stations within the study area. The runoff and meteorological data underwent strict quality control to ensure reliability. If the missing days of a station are less than five, linear interpolation was used to correct the data, while the missing days are more than five, the multi–year average value of other stations on the same day was used.

#### Reservoir data

There are 82 large and medium-sized reservoirs with a total storage capacity of 90 × 10^8^ m^3^. The Table 1 below has a listing of the information on the eight large reservoirs in the MRB. The reservoir capacity coefficient is the ratio of the total capacity of a reservoir to the annual runoff of the basin. It can be used to evaluate the regulation capacity of the reservoir and its impact on the river. The larger the reservoir capacity coefficient, the stronger the regulation capacity of the reservoir.

**Table 1:** Information on the large reservoirs in MRB.

Catchment	Name	Year of completion	Catchment area/(km^2^)	Reservoir capacity/(10^8^m^3^)
Jianxi	Dongxi	1986	554	1.02
Futunxi	Chitan	1980	4766	8.70
Shaxi	Ansha	1978	5184	7.40
Mainstream	Gutianxi	1971	1697	6.24
	Shaxikou	1989	25,562	2.40
	Shuidong	1994	3785	1.10
	Shuikou	1996	52,438	26.00
	Jiemian	2007	1604	18.24

Note. Reservoirs with a total capacity greater than 1.0 × 10^8^ m^3^ are classified as large reservoirs.

#### Actual evapotranspiration data

The Penman-Monteith (P-M) equation was applied to calculate the potential evapotranspiration (PET) of the MRB, which is recommended by the Food and Agriculture Organization of the United Nations (FAO). This method fully considers various factors such as the hydrological cycle, aerodynamic resistance, and energy balance, and its calculation results compare well with measured values^26^. The PET obtained using the P-M equation is corrected using the dual crop coefficient method to determine the actual evapotranspiration (ETa) for the MRB.

#### Circulation indices

Different types of droughts in southern China are closely related to large–scale climate patterns including ENSO, Pacific Decadal Oscillation (PDO), NAO, Southern Oscillation Index (SOI), and Global Mean Land-Ocean Temperature (GMLOT)^27–29^. Therefore, this study selects these three circulation indices for the analysis. The corresponding data were obtained from the NOAA Earth System Research Laboratory (NOAA/ESRL).

### Methods

#### Drought indices

This study uses the SPEI index developed by Vicente-Serrano et al.^30^ to characterize MD. The main parameter for calculating SPEI is the difference between precipitation and PET values, constructing a precipitation evapotranspiration anomaly series. HD is evaluated based on the SRI proposed by Shukla and Wood^31^. The calculation of SRI is fitted using the gamma probability distribution $\:\text{G}\left(\text{x}\right)$. The calculation processes for the SPEI and SRI are as related literature^29,30^.

The obvious studies have shown that the 3–month SPEI/SRI can reflect seasonal drought conditions, which are closely related to agricultural drought^32^. Therefore, in this paper, the SPEI3/SRI3 of May, August, November, and the following February respectively represent spring (March to May), summer (June to August), autumn (September to November), and winter (December to February). The classification of drought events is conducted according to the standards presented in Table 2^29^.

**Table 2:** Wet and drought classification based on SPEI/SRI.

Value range	Classification	Value range	Classification
≥2.0	Extremely wet	−1.0 to − 0.5	Slightly dry
1.5 to 2.0	Severely wet	−1.5 to − 1.0	Moderately dry
1.0 to 1.5	Moderately wet	−2.0 to − 1.5	Severely dry
0.5 to 1.0	Slightly wet	≤−2.0	Extremely dry
−0.5 to 0.5	Normal		

#### Statistical methods

The Mann–Kendall (M–K) trend test is a common non–parametric test recommended by the World Meteorological Organization^33^. Missing data values have negligible influence on the computation of the test results, and the test is not affected by sample values and distribution types. It is typically employed to predict long–term trends in hydro–meteorological time series data such as precipitation, runoff, and temperature^34^. We employed the M–K test to analyze the MD and HD time series in different seasons of the MRB.

#### Analysis of the drought propagation relationship

The Pearson correlation coefficient (PCC) is employed to quantify the correlation between the SPEI and SRI. We use the maximum PCC (MPCC) accumulation period between SPEIn (where n represents the time scale (month), *n* = 1, 2, …, 12) and SRI1 to represent the DPT. This method can effectively display the response intensity and propagation time between different drought types and is widely recognized as a successful approach^35^. The PCC (r) is calculated as follows:

1 $$\:{\text{r}} = \frac{{\sum\limits_{{i = 1}}^{n} {\left( {X_{i} - \mathop X\limits^{ - } } \right)\left( {Y_{i} - \mathop Y\limits^{ - } } \right)} }}{{\sqrt {\sum\limits_{{i = 1}}^{n} {\left( {X_{i} - \mathop X\limits^{ - } } \right)^{2} } } \sqrt {\sum\limits_{{i = 1}}^{n} {\left( {Y_{i} - \mathop Y\limits^{ - } } \right)^{2} } } }},\:i = 1,\:2,\:\: \ldots \:,\:n$$

where $\:\text{n}\:$is the sample size; $\:{X}_{i}$ and $\:{Y}_{i}$ are the corresponding observed values of $\:X$ and $\:Y$; and $\:\stackrel{-}{X}$ and $\:\stackrel{-}{Y}$ are the sample means. The value of $\:\text{r}$ ranges between − 1 and 1: a larger absolute value indicates a higher degree of correlation; $\:\text{r}>0$ indicates a positive correlation; $\:\text{r}<0$ indicates a negative correlation; and $\:\text{r}=0$ indicates no linear correlation.

#### Multivariate regression analysis model

Multiple regression analysis is a method used to quantitatively describe the linear dependence between a dependent variable and multiple independent variables through a regression equation. It is employed to analyze the quantitative relationships between each variable and the dependent variable under linear correlation, thereby determining the degree to which each independent variable influences changes in the dependent variable^36^. Due to variations in the data ranges, conducting multiple regression analysis directly may introduce errors. Therefore, the dependent variables (SPEI/SRI) and independent variables—including temperature, precipitation, and ETa data—were first standardized to ensure that all data sequences involved in the analysis fell within the range of [0, 1]. The Min-Max normalization method was applied for standardization, calculated using the following formula:

2 $$\:{y}_{i}=\frac{{x}_{i}-\text{min}\left({x}_{j}\right)}{\text{max}\left({x}_{j}\right)-\text{min}\left({x}_{j}\right)}\:$$

Where $\:{\text{x}}_{\text{i}}$ represents the data point in the sequence that requires standardization, $\:{\text{x}}_{\text{j}}$ denotes the entire dataset, new sequence $\:{\text{y}}_{1}$, $\:{\text{y}}_{2}$, …, $\:{\text{y}}_{\text{n}}$ ∈ [0, 1].

After standardization, multiple regression analysis was performed for each independent variable, and the regression coefficients of the standardized data series were determined based on the least squares method. The calculation formula for multiple regression is as follows:

3 $$\:{y}_{i}=a+{bx}_{i1}+{cx}_{i2}+\dots\:+{gx}_{in}$$

where $\:{x}_{i1}$, $\:{x}_{i2}$, …, $\:{x}_{in}$ as the independent variable; $\:a$, $\:b$, …, $\:g\:$as the regression coefficient.

Finally, the relative contribution of each independent variable to the variation in the dependent variable was calculated using the regression coefficients according to the following formula. The relative contribution rate refers to the proportion of change attributed to a specific influencing factor during SPEI/SRI variation, representing the degree to which SPEI/SRI changes respond to that factor over the entire study period. The calculation formula is as follows:

4 $$\:{\mu\:}_{1}=\frac{\left|a\right|}{\left|a\right|+\left|b\right|+\left|c\right|+\dots\:}$$

where $\:\text{a}$, $\:\text{b}$, $\:\text{c}$…represent the standardized regression coefficients of the data sequence, the relative contribution rate of $\:{{\upmu\:}}_{1}$ to the change of $\:{\text{Y}}_{\text{s}}$ when $\:{\text{X}}_{1}$ changes.

## Results

### Characteristics of MD and HD at different time scales

**Fig. 2:**
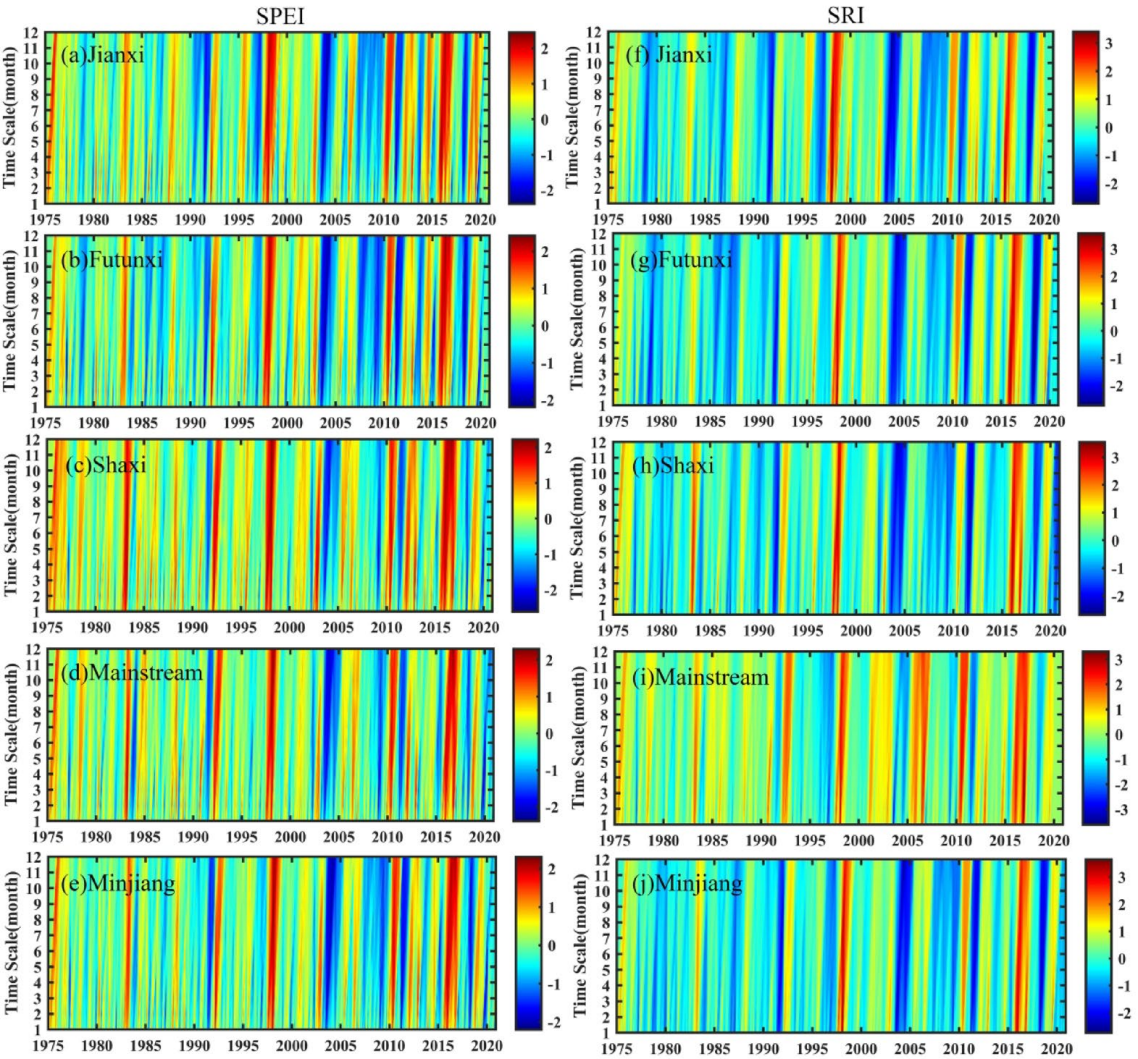
Temporal variation of SPEI and SRI at different time scales during 1975–2020.

Figure 2 presents the alternating dry and wet characteristics in the MRB. The spatial and temporal trends of MD and HD were similar, with a strong correlation between them. From 1975 to 2000, the MRB exhibited a drying trend, with SPEI and SRI decreasing at rates of 0.041/10a and 0.022/10a, respectively. The SPEIn and SRIn did not change significantly, with only one extreme wet event occurring in 1998 and no obvious extreme drought events. After 2000, the drying trend reversed, with SPEI and SRI increasing at rates of 0.127/10a and 0.155/10a, indicating a trend toward wetter conditions. However, both types of drought events occurred frequently, including three drought events of higher severity. HD generally lags behind MD, and HD has a higher severity and a longer duration than MD. For example, the most severe MD occurred in July 2003 with the SPEI of − 2.1 and lasted for 5 months, followed by an extreme HD with the SRI of − 2.47 in August 2003 and lasted for 15 months. Moreover, the severity of the MD and HD gradually intensified as the time scale increased from 1 to 12 months. For extreme drought events occurring during 2003–2004, the SPEI values at the 1, 3, 6, and 12–month scales were − 0.35, − 0.58, − 0.82, and − 2, respectively, while the corresponding SRI values reached − 0.95, − 1.31, − 1.47, and − 2.75.

The subbasins in MRB exhibited different trends of wetting and drying on the annual and seasonal scales from 1975 to 2020 (Table 3). At the annual scale, the Futunxi and mainstream subbasins tended towards meteorological drying but showed hydrological wetting trends, with insignificant changes. At the seasonal scale, all subbasins experienced meteorological and hydrological drought in spring, while SPEI in the Shaxi, Mainstream and Minjiang river basins showed significant trends, and only the SRI in the Shaxi basin reached the significant level (*p* < 0.05). In summer, all subbasins showed insignificant meteorological wetting, while the Shaxi and mainstream subbasins experienced hydrological drying. In autumn, all subbasins suffered meteorological drought and only Shaxi subbasin showed hydrological drought. In winter, it showed meteorological drought in Shaxi and Mainstream subbasins, while hydrological humidification occurred significantly (*p* < 0.01).

**Table 3:** Annual and seasonal Z-value trends analysis of SPEI and SRI during 1975–2020.

Catchment		Annual	Spring	Summer	Autumn	Winter
Jianxi	SPEI	0.09	−1.33	1.36	−0.81	0.60
	SRI	0.09	−1.75	0.61	0.31	1.09
Futunxi	SPEI	−0.19	−1.53	1.34	−0.62	0.03
	SRI	0.47	−1.41	0.75	1.1	1.81
Shaxi	SPEI	−1.16	−2.42*	1.29	−1.14	−0.01
	SRI	−1.18	−2.55*	−0.5	−0.19	2.45*
Mainstream	SPEI	−1.46	−2.23*	1.42	−1.17	−0.17
	SRI	0.01	−1.75	−0.22	1.77	3.31**
Minjiang	SPEI	−0.34	−2.03*	1.33	−0.97	0.13
	SRI	−0.15	−1.91	0.34	0.78	2.49*

* and ** denote significance at the 0.05 and 0.01 levels, respectively.

### Influential factors of MD and HD

Table 4 displays the multiple regression coefficients and relative contribution rates between seasonal SPEI/SRI and climatic factors. The results indicated that precipitation showed a significant positive correlation with SPEI in all four seasons (*p* < 0.01), and temperature always exhibited a significant negative correlation with SPEI (*p* < 0.01), while the correlation between ETa and SPEI was insignificant. Moreover, the relative contribution rate of precipitation on SPEI in all climate factors exceeded 80%, indicating that precipitation was the primary factor affecting MD in all different seasons. Regarding the factors influencing HD, precipitation demonstrated a significant positive correlation with SRI in all four seasons, with a relative contribution rate on HD exceeding 80% for winter. The significant negative impact of temperature on SRI only occurred in summer (*p* < 0.05). There was also no significant correlation between ETa and SRI.

**Table 4:** Multivariate regression coefficients and relative contribution rate between SPEI/SRI and Climatic factors in four seasons.

Season	Climatic factor	Multivariate regression coefficients		Relative contribution rate/%	
		SPEI	SRI	SPEI	SRI
Spring	Precipitation	1.025**	0.828**	88.29	66.83
	Temperature	−0.133**	−0.253	11.46	20.42
	ETa	−0.003	−0.158	0.26	12.75
Summer	Precipitation	0.928**	0.881**	89.49	67
	Temperature	−0.102**	−0.425*	9.84	32.32
	ETa	0.007	−0.009	0.68	0.68
Autumn	Precipitation	0.991**	0.657**	83.56	63.23
	Temperature	−0.178**	0.015	15.01	1.45
	ETa	−0.017	−0.367	1.43	35.32
Winter	Precipitation	0.962**	0.854**	82.58	82.19
	Temperature	−0.106**	0.096	9.10	9.24
	ETa	−0.097	0.089	8.33	8.57

* and ** denote significance at the 0.05 and 0.01 levels, respectively.

**Fig. 3:**
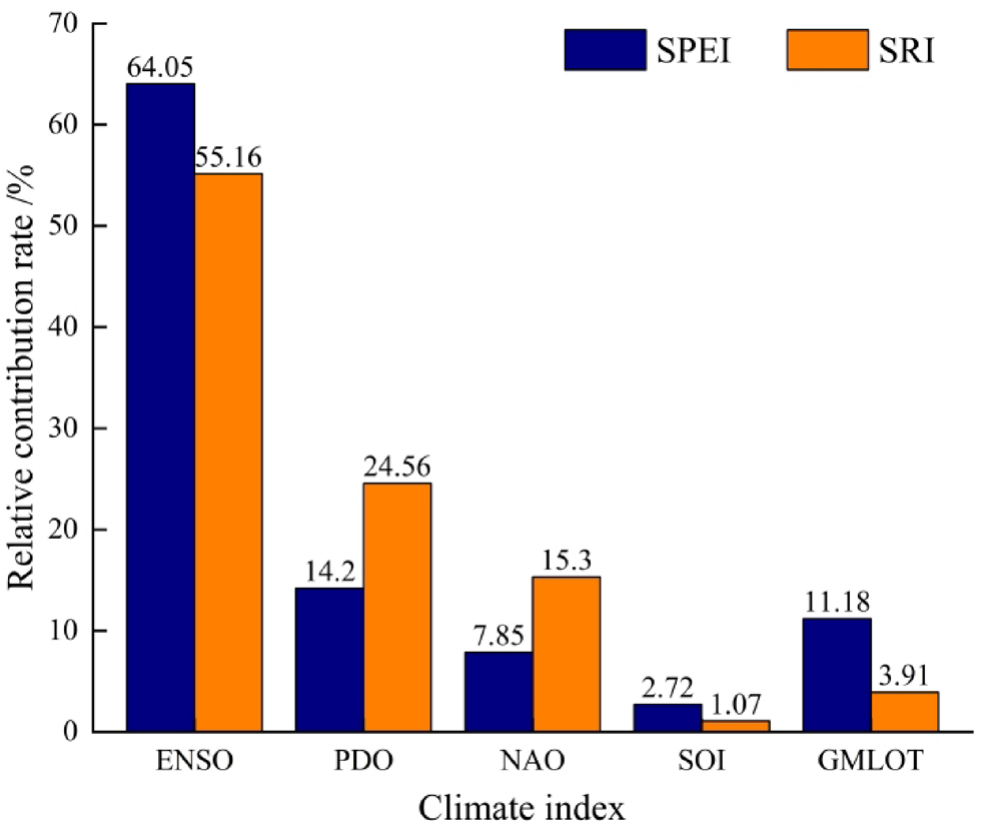
The relative contribution rate of large-scale circulation factors to the influence of SPEI and SRI.

Large–scale circulation factors influence MD and HD processes by modulating key climatic processes such as regional moisture transport, precipitation distribution, and surface energy balance^16,37^. Figure 3 presents the relative contribution rates of different climatic indices to MD and HD, revealing distinct impacts of these indices on drought variability. Notably, ENSO exhibited the highest contribution rates to both SPEI and SRI, reaching 64.05% and 55.16%, respectively, indicating its dominant role in driving drought variability in the MRB. The PDO also demonstrated significant contributions, accounting for 24.56% of SPEI and 14.2% of SRI variability. The contribution rate of GMLOT to SPEI can reach 11.18%, while the contribution rate of NAO to SRI can reach 15.3%. In contrast, the impact of SOI is relatively small, with contribution rates to SPEI and SRI reaching only 2.72% and 1.07%, respectively.

### Spatial differences in drought propagation across different seasons

**Fig. 4:**
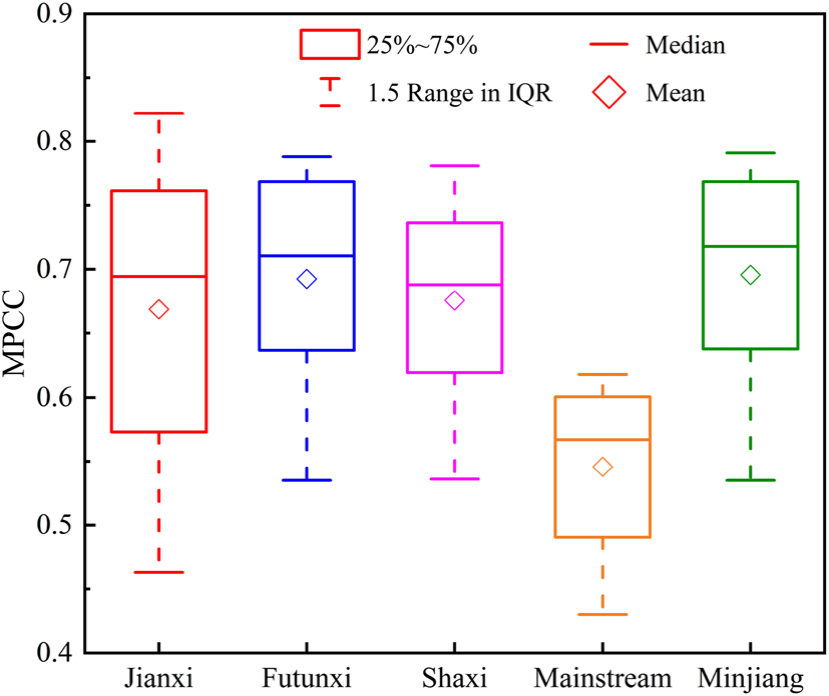
PCC between SPEI1–12 and SRI1 from 1975 to 2020.

Table 3 reveals the lack of synchronicity in the SPEI and SRI on the seasonal scale, indicating a certain lag between MD and HD. To quantify the characteristics of the propagation from MD to HD, the MPCC between SPEIn (*n* = 1–12) and SRI1 was used to represent the DPT. Figure 4 shows that the MPCC for MD and HD in each subbasin of the MRB ranged from 0.48 to 0.79, indicating an extremely strong response relationship between MD and HD. However, there is the spatial heterogeneity involved. The MPCC exhibited the highest value of 0.79 in the Futunxi subbasin, while the lowest value of 0.48 occurred in the mainstream basin. The MPCC values in the Jianxi and Futunxi subbasins were higher than those of other subbasins, indicating a closer relationship between MD and HD in these two subbasins. As shown in Table 5, the DPT of the MRB mainly with an average of 3 months. In the Jianxi subbasin, the MPCC occurred at 2 months with the value of 0.822, indicating that the DPT was generally 2 months. The propagation speed in the mainstream subbasin was relatively slower, with the value of MPCC reaching 0.618, and the highest correlation occurred at 5 months. Both the Shaxi and Futunxi subbasins exhibited a DPT of 3 months.

**Table 5:** The DPT and MPCC in each subbasin.

	Jianxi	Futunxi	Shaxi	Mainstream	Minjiang
DPT/mon	2	3	3	5	3
MPCC	0.822	0.788	0.781	0.618	0.791

**Fig. 5:**
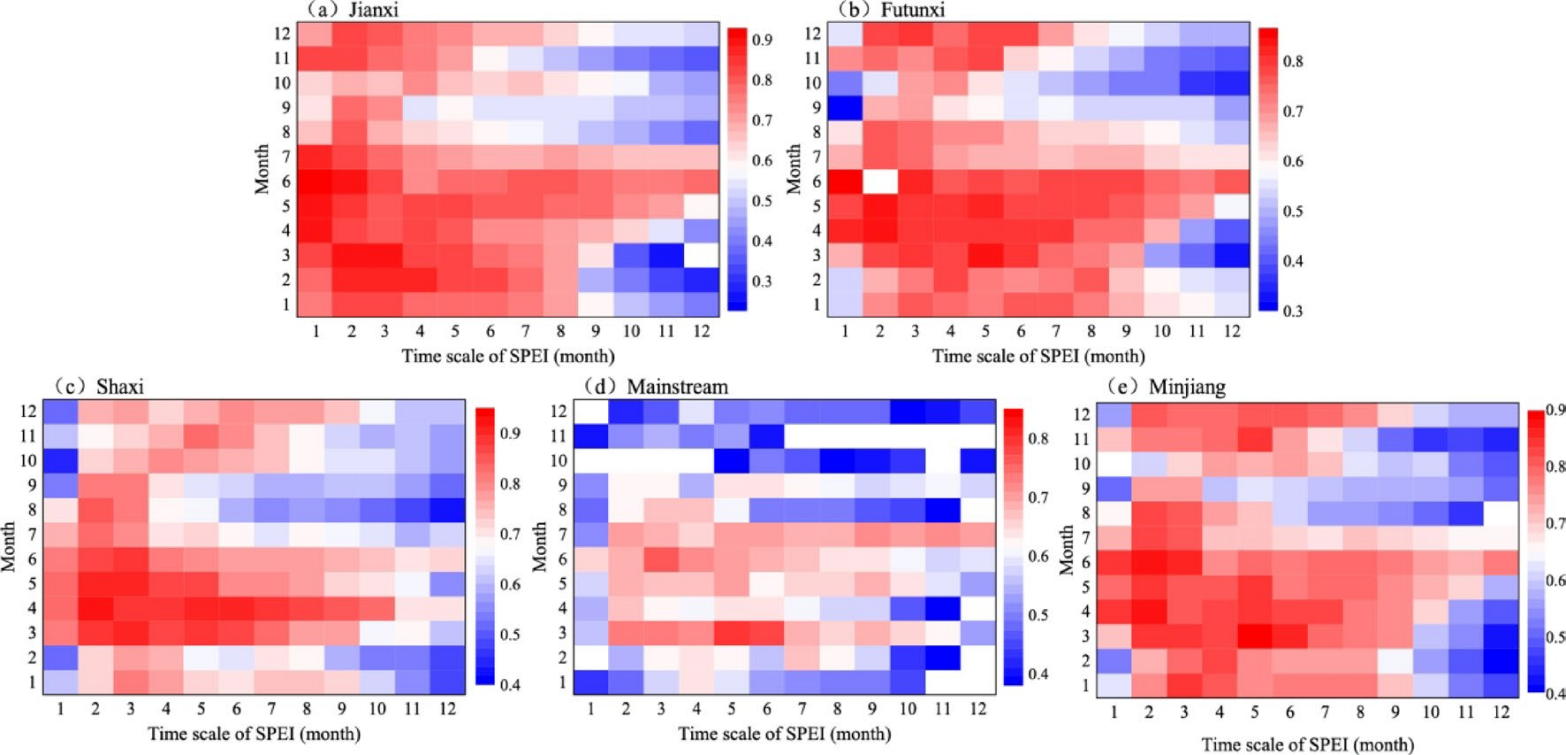
Correlation coefficients between SPEI1–12 and SRI1.

The hydrological cycle exhibits different characteristics in different seasons, therefore, the propagation of MD to HD varies considerably with seasons. Figure 5 presents the correlation coefficients between the SPEI on a 1–12 month timescale and the SRI on a 1–month timescale in the MRB. The DPT from MD to HD in each subbasin has distinct seasonal characteristics. The high correlation coefficients (> 0.7) of the Jianxi subbasin were mainly concentrated in spring and summer, with the DPT from MD to HD determined as 2 months in spring and autumn, 3 months in winter, and 1 month in summer. The Futunxi subbasin had a DPT of 3–5 months in autumn and winter, and 2 months in spring and summer. The Shaxi subbasin had a DPT of 5 months in autumn, 3 months in summer and winter, and 2 months in spring. The DPT in the mainstream subbasin reached 6–7 months in autumn and winter, 3 months in summer, and 5 months in spring. The MRB had a DPT of 3–5 months in autumn and winter, and 2 months in spring and summer. Overall, the DPT of MD and HD in each subbasin of the MRB is relatively shorter in spring and summer, and longer in autumn and winter.

## Discussion

### Evolution characteristics and influencing factors of drought in the MRB

From 1975 to 2020, the MRB exhibited largely consistent trends in MD and HD, reflecting a typical drought response pattern in humid regions under global warming. This aligns with observations in other humid monsoon regions, such as the Mekong River Basin in Southeast Asia^5^ and the Amazon Basin in South America^38^. The increased drought frequency in the MRB after 2000 supports the conclusion of IPCC that enhanced precipitation variability in humid regions leads to more frequent drought events^39^. Despite the potential increases in annual mean precipitation, the rising temperatures and intensified evapotranspiration will exacerbate drought conditions. Although the MRB shows an insignificant drought trend at the annual scale, the opposing seasonal shifts between wet and dry conditions are reshaping regional water balance patterns. This asymmetric response closely matches observations in other humid regions of southern China^40^suggesting that many humid areas worldwide may face a new drought risk characterized by increased total precipitation but imbalanced seasonal distribution.

Most studies considered that climatic conditions (precipitation, temperature, and ETa) have a great impact on drought^14,16^. Our research also found that insufficient precipitation can largely contribute to explain over 80% of meteorological and hydrological drought in different seasons. Over the past 46 years, spring precipitation has decreased at a rate of − 9.775 mm/10a, while temperature and ETa have increased at the rate of 0.271 ℃/10a and 4.285 mm/10a in the MRB, respectively. Runoff is primarily replenished by rainfall, and a decrease in rainfall directly leads to reduced runoff. The increase in temperature and ETa consumes a large amount of water, which, together with insufficient precipitation, leads to soil moisture deficits, lowering SPEI and SRI values, thereby triggering or exacerbating spring drought^2^. Furthermore, multiple atmospheric circulation factors also play an important role in drought in the MRB. Atmospheric circulation can indirectly affect drought by influencing regional climate through teleconnections and perturbations, and this makes the impact of atmospheric circulation on drought less than meteorological factors^41^. ENSO is the key factor affecting MD and HD (Fig. 3), which can be attributed to the multidecadal oscillatory relationship between precipitation in southern China and ENSO events^42^. Previous studies have confirmed that variations in large-scale circulation factors such as ENSO modulate regional precipitation, evapotranspiration, temperature, and other meteorological variables^27–29^. These climatic changes are recognized as one of the primary causes of streamflow instability in river basins, thereby driving the evolution mechanisms of MD and HD. Fu et al.^43^ determined ENSO as the most significant factor affecting precipitation, with the strongest positive effect in southern China and coastal areas. It is reported that the northeasterly wind anomalies during and after ENSO prevent vapor transport from East Asian Summer Monsoon (EASM), which results in the development of droughts^44^.

**Fig. 6:**
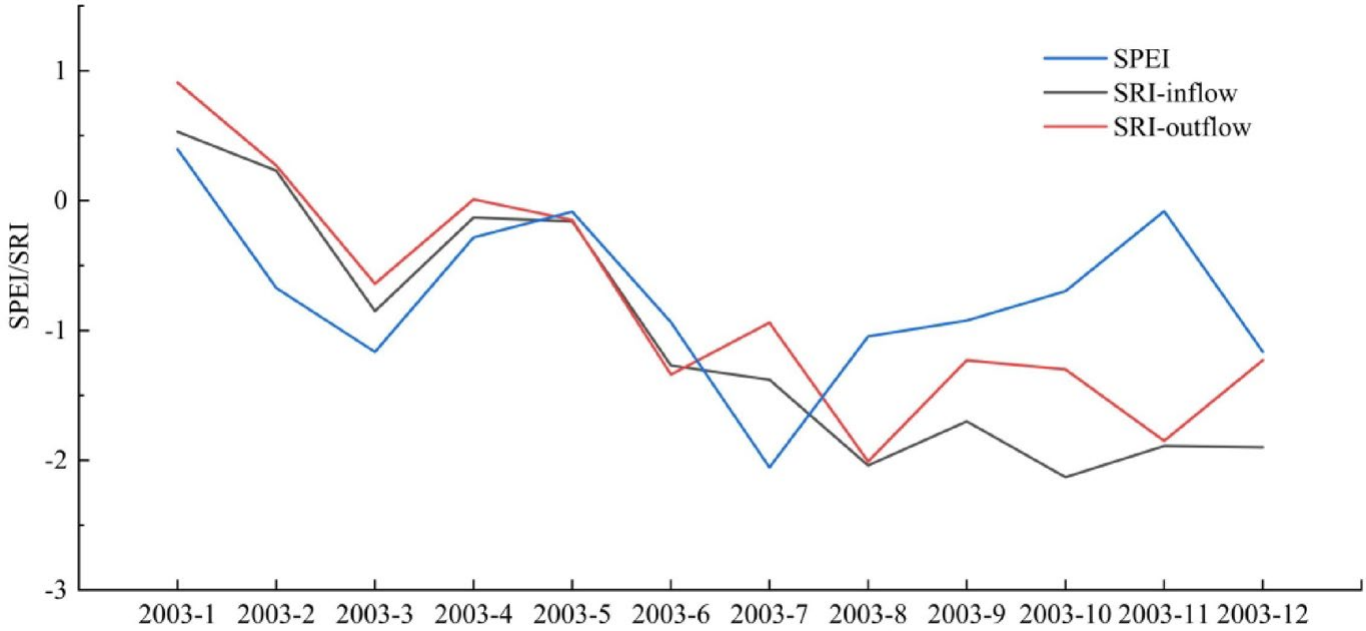
SPEI of the mainstream and SRI of inflow/outflow of Shuikou Reservoir in 2003.

In addition to climatic conditions, human activities also affect the variation of dry and wet characteristics. It is presented that the trend of hydrological humidification occured in autumn and winter with less precipitaion in the MRB, which may be related to the reservoir regulation. Due to 2003 being the driest year in the MRB (Fig. 2), the observed data of inflow and outflow of Shuikou Reservoir in 2003 were selected to calculate SRI values, so as to analyze the impact of reservoir operation on drought event in dry year. Figure 6 shows that SRI valules of outflow are generally higher than those of inflow in Shuikou Reservoir in 2003, especially in dry seasons. During the autumn and winter (September, October, and December), the SRI values at the time of inflow were − 1.7, − 2.13, and − 1.9, respectively, indicating severe HD. However, at the time of outflow, the SRI values were − 1.23, − 1.3, and − 1.23, respectively, indicating only moderate HD. This demonstrated that Shuikou Reservoir released more water into the downstream of Minjiang River, which could mitigate the severity of HD to some extent during the dry season. Due to the lower precipitation in autumn and winter, the reservoir released water during the dry season to meet the downstream water demand. This process not only alleviates drought but also increases the replenishment of rivers and groundwater, thereby significantly enhancing autumn and winter hydrological wetting^45^. Liu et al.^18^ also argued that the regulation effect of reservoirs reduces autumn and winter HD in downstream areas.

### Propagation time of drought and its influencing factors

The duration of DPT exhibited significant regional variations, which were closely related to climatic conditions^11^. A comparative analysis of multiple river basins worldwide reveals that humid climate zones generally exhibit shorter DPT (1–6 months), while arid/semi-arid regions tend to have relatively longer DPT (3–8 months)^7^. The spatial pattern of DPT in China aligns with this global trend, showing a gradual decrease from the arid northwest (3–8 months) to the humid southeast (1–5 months)^46,47^a distribution strongly linked to regional variations in hydrothermal conditions. In humid regions worldwide, similar DPT ranges are observed, including the Yangtze River Basin (2–4 months)^48^the Indus River Basin (2–4 months)^8^the Mediterranean region (4 months)^49^and the MRB in this study (averaging 3 months). Notably, although the MRB is generally humid, its upper DPT limit (7 months) is slightly longer than other humid basins, possibly due to localized factors such as human activities. Moreover, the propagation from MD to HD varies with the time scale. In spring and summer, when the temperature is high, the DPT is shorter and the response rate is faster. However, in autumn and winter, with lower temperatures, the DPT is longer and the response rate is slower. This is attributed to the location of the MRB within the monsoon zone in the east of China, where temperature and precipitation coincide with the rainy and warm seasons. In the hot season, especially in summer—which is also in the flood season—heavy and torrential rain events often occur. Sufficient precipitation combined with high temperatures in summer accelerates the water cycle^37^. This increases the sensitivity of MD propagation to HD to some extent, resulting in a faster response of summer runoff to precipitation compared to other seasons^10,15^. Gevaert et al.^10^ also concluded that the DPT is faster during the hot season and slower during the cold season. Conversely, when the rainy season is over, under the influence of the Pacific subtropical high pressure, precipitation within the basin decreases and is prone to drought. This reduces the sensitivity of MD propagation to HD in autumn and winter, and consequently, extends the DPT.

The responses of MD and HD in the MRB are not entirely consistent across subbasins. As shown in Table 3, four subbasins exhibited a trend of meteorological wetting in summer, while Shaxi and the mainstream subbasins showed a trend of hydrological drying. In autumn, all subbasins exhibited a trend of meteorological drying, but Jianxi, Futunxi, and the mainstream subbasins showed hydrological wetting. In winter, Shaxi and the mainstream subbasins experienced meteorological drying but experienced significant hydrological wetting (*p* < 0.05). The unsynchronized trends of MD and HD are related to the difference in reservoir regulation capacity in different subbasins. Numerous studies have shown that major human activities, such as large–scale hydraulic projects, have a significant impact on the seasonal drought propagation within basins^12,18^. Reservoirs typically allevaite seasonal floods and droughts by storing more water and releasing less water during wet periods, and releasing more water while storing less during dry periods^21^. During the wet seasons, water storage reduces discharge, potentially causing drought downstream. During the dry seasons, the reservoir drainage can alleviate drought and even lead to wetness^48^.

**Fig. 7:**
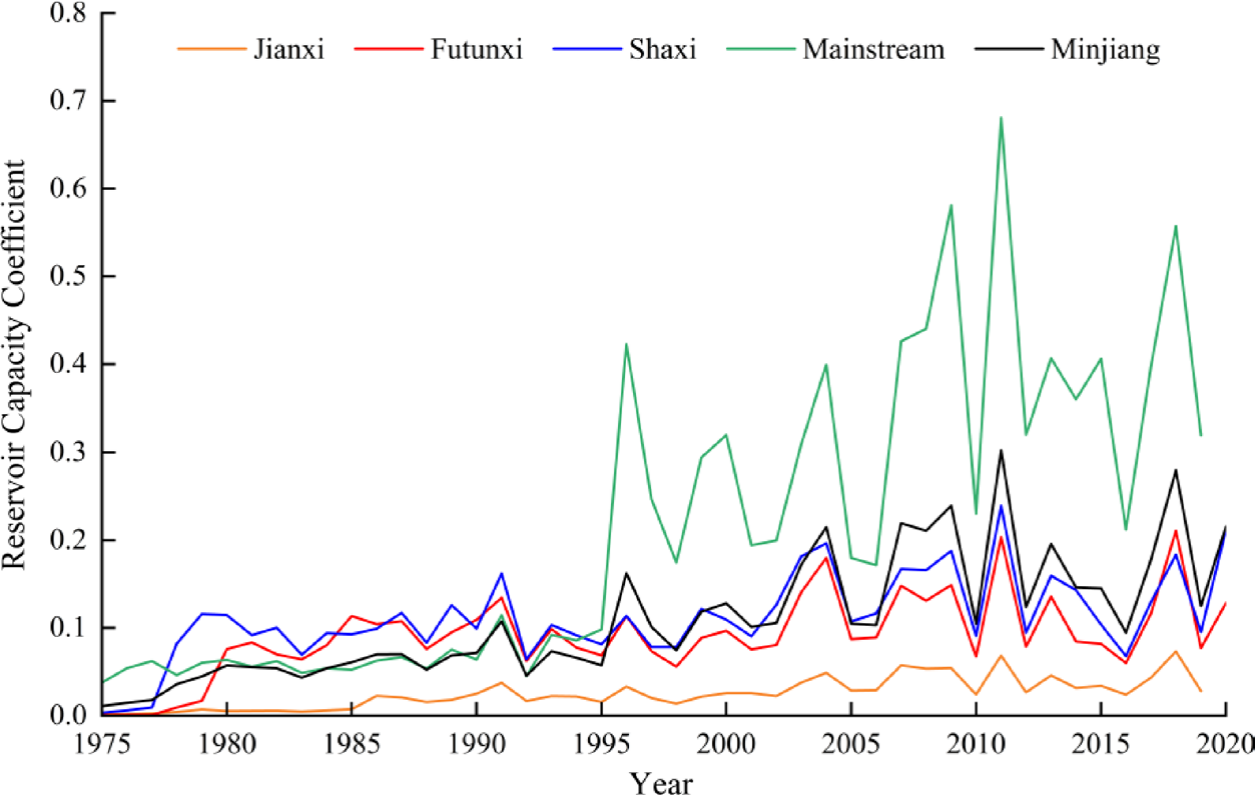
The variation trend of reservoir capacity coefficient during 1975–2020.

Figure 7 shows the trend of the reservoir capacity coefficient in the MRB from 1975 to 2020. It can be observed that the proportion of reservoir storage to runoff in each basin is increasing, indicating that the reservoir regulation capacity is gradually enhancing. This increases runoff during the dry season and reduces it during the rainy seasons, thereby altering the response relationship of HD to MD. The interannual variation trends of the reservoir capacity coefficients for Jianxi, Futunxi, Shaxi, the mainstream, and the Minjiang are 0.011/10a, 0.02/10a, 0.022/10a, 0.105/10a, and 0.041/10a, respectively. Among these, the trend in the mainstream is the most significant, with a notable change occurring in 1996. This is primarily due to the completion and commencement of impoundment of the Shuikou Reservoir in 1996. With a maximum capacity of 26 × 10^8^ m^3^Shuikou Reservoir is the largest hydropower station in southeastern China. Therefore, the study period is divided into 1975–1995 and 1996–2020 to compare the DPT in these two periods and analyze the impact of reservoir regulation on seasonal DPT. Table 6 shows that the DPT in the four seasons from 1975 to 1995 were 2, 1, 2, and 2 months, respectively, and the corresponding values from 1996 to 2000 were 6, 3, 5, and 7 months, respectively. Thus, the operation of large reservoirs has extended the DPT in the four seasons by 4, 2, 3, and 5 months, respectively. It is consistent with previous studies that reservoirs can extend drought propagation^50^. Xing et al.^51^ also pointed out that reservoirs exacerbate downstream HD in summer, alleviate HD in winter, reduce the correlation between MD and HD during wet periods, and increase the duration and severity of HD events during flood season.

**Table 6:** The drought propagation time before and after reservoir impact (unit: month).

Year	Spring	Summer	Autumn	Winter
1975–1995	2	1	2	2
1996–2020	6	3	5	7

### Limitations

Our study quantifies the propagation time from MD to HD using drought indices and analyzes the factors influencing the DPT. The maximum Pearson correlation coefficient method applied here to determine the duration of drought propagation have been adopted widely. Luo et al.^24^ used both maximum Pearson correlation coefficient (MPCC) and Convergent Cross Mapping (CCM) to investigate the propagation characteristics from meteorological to hydrological drought in nine major river basins in China. The results showed that in humid areas such as the Pearl River Basin and the Southwest River Basin in south of China, where runoff changes were basically only affected by precipitation, correlation could be used as a substitute to explore drought propagation time. In our study of the humid region of the MRB, despite the influence of various climatic conditions and human activities, the linear relationship between rainfall and runoff remains very significant. Therefore, the maximum correlation method is suitable to measure propagation time in MRB. However, the traditional Pearson correlation coefficient mainly reflects the statistical correlation between variables, but this correlation may not necessarily reveal potential causal mechanisms^52^. The relationship between meteorological and hydrological drought propagation maybe nonlinear. Wu et al.^53^ recommended the run theory-based approach rather than the correlation analysis to calculate DPT. Luo et al.^24^ proposed that convergent cross mapping can better elucidate the dynamic mechanisms of drought propagation by capturing causal relationships between variables. Therefore, we can survey the calculation of drought propagation time from a nonlinear perspective in the future and further explore the mechanistic characteristics of drought propagation.

Furthermore, due to the lack of more detailed information of large reservoirs in the MRB, only the inflow and outflow of Shuikou Reservori in 2003 was obtained as a typical drought year to qualitatively discuss the impact of reservoir operation on drought. Additionally, we did not consider other human activities, including irrigation, water extraction, land use and cover changes. In some regions, the impact of human activities on drought propagation may be greater than natural factors^4^. For example, Wossenyeleh et al.^54^ showed that artificial groundwater extraction can alter the hydrological conditions of wetlands, exacerbating groundwater drought in wetlands and accelerating drought propagation. Agricultural irrigation can extend the DPT during the rainy season, while domestic water supply and urban area expansion can shorten it during the dry season^55^. Therefore, we need to increase the representative indicators of different human activities and quantify the impact of individual factors on MD and HD. However, due to the uncertainty of human activities, their impact on drought propagation and the mechanisms of large–scale drought propagation have not been fully resolved. Our future research will continue to focus on these issues.

## Conclusions

This study analyzed MD and HD in MRB from 1975 to 2020 using SPEI and SRI at different time scales. PCC was used to explore the relationship between MD and HD in terms of propagation, and potential factors affecting DPT were discussed. The main conclusions are as follows:

HD in the MRB lags behind MD and lasts longer. The overall DPT with an average of 3 months. The DPT in each subbasin gradually extends from upstream to downstream areas, and the DPT in spring and summer is generally shorter than that in autumn and winter. MD and HD in the MRB are influenced by meteorological conditions, and large–scale climate patterns. Precipitation and ENSO are the main meteorological and circulation factors that affect drought. During drought propagation, reservoir regulation significantly alters the response pattern of HD to MD and extends the DPT of the four seasons after 1996 by 4, 2, 3, and 5 months, respectively.

The findings of this study quantitatively analyzes the propagation time from MD to HD at different time scales in the MRB, providing a preliminary understanding of the basin’s drought propagation mechanisms. The results help to reveal the drought propagation process, enhance drought monitoring and prevention, and provide useful and valuable information for local drought resistance and disaster reduction.

## Data Availability

The data that support the findings of this study are available from the China Meteorological and Hydrological Administration but restrictions apply to the availability of these data, which were used under license for the current study, and so are not publicly available. Data are however available from the authors upon reasonable request.
